# Impaired lymphocyte trafficking in mice deficient in the kinase activity of PKN1

**DOI:** 10.1038/s41598-017-07936-9

**Published:** 2017-08-09

**Authors:** Rana Mashud, Akira Nomachi, Akihide Hayakawa, Koji Kubouchi, Sally Danno, Takako Hirata, Kazuhiko Matsuo, Takashi Nakayama, Ryosuke Satoh, Reiko Sugiura, Manabu Abe, Kenji Sakimura, Shigeharu Wakana, Hiroyuki Ohsaki, Shingo Kamoshida, Hideyuki Mukai

**Affiliations:** 1 Graduate School of Medicine, Kobe University, Kobe, 650–0017 Japan; 2 Center for Innovation in Immunoregulative Technology and Therapeutics, Kyoto University Graduate School of Medicine, Kyoto, Japan; 3 Graduate School of Science and Technology, Kobe University, Kobe, 657-8501 Japan; 4 Department of Fundamental Biosciences, Shiga University of Medical Science, Seta-Tsukinowa-cho Otsu, Shiga, 520-2192 Japan; 5 Division of Chemotherapy, Kindai University School of Pharmacy, Kowakae, Higashi-Osaka 577-8502 Japan; 6 Laboratory of Molecular Pharmacogenomics, School of Pharmaceutical Sciences, Kindai University, 3-4-1, Kowakae, Higashi-Osaka 577-8502 Japan; 7 Department of Cellular Neurobiology, Brain Research Institute, Niigata University, Niigata, 951-8585 Japan; 8 Japan Mouse Clinic, RIKEN BioResource Center, 3-1-1 Koyadai, Tsukuba-shi, Ibaraki 305-0074 Japan; 9 Laboratory of Pathology, Department of Medical Biophysics, Kobe University Graduate School of Health Sciences, 7-10-2 Tomogaoka, Suma, Kobe, Hyogo 654-0142 Japan; 10 Biosignal Research Center, Kobe University, Kobe, 657-8501 Japan

## Abstract

Knock-in mice lacking PKN1 kinase activity were generated by introducing a T778A point mutation in the catalytic domain. PKN1[T778A] mutant mice developed to adulthood without apparent external abnormalities, but exhibited lower T and B lymphocyte counts in the peripheral blood than those of wild-type (WT) mice. T and B cell development proceeded in an apparently normal fashion in bone marrow and thymus of PKN1[T778A] mice, however, the number of T and B cell counts were significantly higher in the lymph nodes and spleen of mutant mice in those of WT mice. After transfusion into WT recipients, EGFP-labelled PKN1[T778A] donor lymphocytes were significantly less abundant in the peripheral circulation and more abundant in the spleen and lymph nodes of recipient mice compared with EGFP-labelled WT donor lymphocytes, likely reflecting lymphocyte sequestration in the spleen and lymph nodes in a cell-autonomous fashion. PKN1[T778A] lymphocytes showed significantly lower chemotaxis towards chemokines and sphingosine 1-phosphate (S1P) than WT cells *in vitro*. The biggest migration defect was observed in response to S1P, which is essential for lymphocyte egress from secondary lymphoid organs. These results reveal a novel role of PKN1 in lymphocyte migration and localization.

## Introduction

Protein kinase N (PKN) is a serine/threonine protein kinase with a catalytic domain homologous to that of protein kinase C (PKC) and a unique regulatory region containing antiparallel coiled-coil (ACC) domains^1, 2^. PKN1, also known as PKNα or PRK1, is one of three PKN isoforms derived from different genes in mammals. PKN1 was first described as a fatty acid- and phospholipid-activated serine/threonine protein kinase and a protease-activated protein kinase^3, 4^. PKN1 is also an effector protein kinase of Rho family GTPases, such as RhoA, RhoB, RhoC, and Rac, in mammalian tissues^1, 5–11^. Various PKN1 functions have been revealed using cell culture experiments; for example, it is involved in the regulation of cytoskeletal reorganization^12, 13^, cell adhesion^14, 15^, cell-cycle regulation^16–18^, and tumorigenesis^19, 20^. At the organismal level, PKN1 is ubiquitously expressed in various mammalian tissues, but is particularly highly expressed in lymphoid organs, suggesting major roles in lymphoid tissues^1^. Yasui *et al*. have reported that PKN1 knockout (KO) mice appear normal and do not exhibit defects in lymphocyte development in PKN1 KO mice within 12 weeks of age^21^. However, they also reported that germinal centers form spontaneously in the spleen at more than 30 weeks of age in PKN1 KO mice, even in the absence of immunization or infection, and these mice eventually develop an autoimmune-like disease characterized by autoantibody production and glomerulonephritis^21^.

PKN1 functions as an intracellular signalling molecule, in some cases independent of phosphorylation activity, e.g., it activates phospholipase D1^22^ and acts as a scaffold protein for the p38γ MAPK signalling pathway^23^. Therefore, to explore the role of the phosphorylation activity of PKN1 *in vivo*, we generated knock-in mice expressing kinase-negative mutant of PKN1 by introducing a T778A point mutation in the activation loop of the catalytic domain. PKN1[T778A] mice grew to adulthood, without apparent external abnormalities, consistent with PKN1 KO mice reported previously^21^. However, PKN1[T778A] mice showed lymphopenia and the accumulation of lymphocytes in secondary lymphoid organs. We discuss the mechanism underlying this phenomenon and the essential role of PKN1 in lymphocyte trafficking *in vivo*.

## Results

### Generation of PKN1 kinase-negative knock-in mice

**Figure 1:**
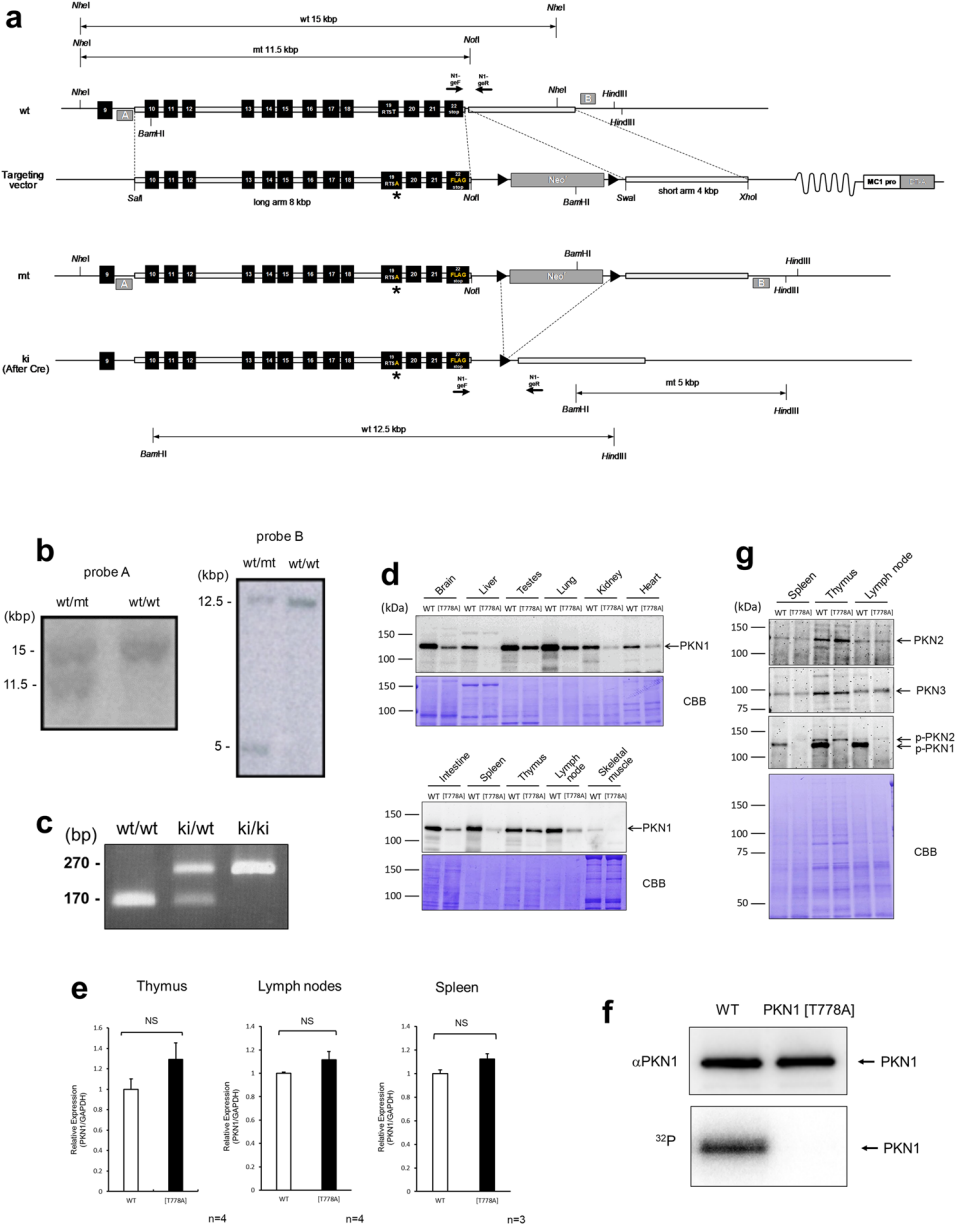
Generation of PKN1[T778A] mice. (**a**) Schematic diagram of the *PKN1* genomic DNA, targeting vector, and disrupted gene. The targeting vector and a partial map of *PKN1* locus before (wt) and after (mt) homologous recombination in embryonic stem cells, and after further deletion of the neomycin resistance cassette (ki) by Cre-mediated recombination. Positions of loxP sites are designated by black triangles. Crosses with heterozygous mice generated homozygous PKN1 kinase-negative knock-in (PKN1[T778A]) mice. Exons are denoted by black boxes. Positions of the genomic DNA probes (A and B) used for Southern blotting and the primers used for discrimination between wt and ki (N1-geF and N1-geR) are indicated. A, probe A; B, probe B; Cre, P1 bacteriophage cyclization recombination; loxP, locus of X-over in P1; E, exon; Neo^r^, neomycin-resistance gene; MC1 pro, MC1 promoter; DT-A, Diphtheria toxin A; *, T778A mutation. (**b**) Southern blot results for representative littermates (F2 mice) obtained by crossing F1 mice with WT mice are shown. Genomic DNA of F2 mice was digested with *Nhe*I and *Not*I, probed with probe A on the left, and digested with *Bam*HI and *Hin*dIII, probed with probe B on the right. (**c**) PCR genotyping. Representative results are shown for discrimination between wt and ki allele (lacking the Neo cassette). (**d**) Whole-cell lysates of each tissue from WT and PKN1[T778A] mice were resolved by SDS-PAGE and subjected to immunoblot analyses using αC6 for PKN1 detection. Coomassie Brilliant Blue (CBB)-stained PVDF membrane is shown (lower panel). (**e**) *PKN1* expression in the thymus, lymph nodes, and spleen of WT and PKN1[T778A] mice was measured by RT-qPCR. Data represent fold changes in *PKN1* gene expression in [T778A] mice normalized to *GAPDH* relative to expression in WT mice. Data were analysed with unpaired *t*-tests. NS, not significant. (**f**) Immunoprecipitation and kinase assay of PKN1. Immunoblotting results using αC6 are shown in the upper panel, and autoradiography results are shown in the lower panel. (**g**) Expression of PKN2 and PKN3, and phosphorylation levels of PKN2 at Thr815 are shown. For raw gel data see Supplementary Fig. 3.

Thr778 in the activation loop of the catalytic domain of mouse PKN1, corresponding to Thr774 of human PKN1, is critical for kinase activity, and an alanine mutation at this position results in a complete loss of kinase activity^24^. To investigate the physiological importance of PKN1 kinase activity, we generated a PKN1 kinase-negative knock-in mouse line by introducing the T778A mutation into the *PKN1* gene. The knock-in vector was introduced into C57BL/6 embryonic stem (ES) cells by electroporation, and chimeric mice were generated from the recombinant ES clone. The PKN1 heterozygous knock-in (PKN1 T778A/+) mouse line was established after removing *neo* (the neomycin-resistance gene) by crosses with EIIα-Cre mice^25^ expressing Cre recombinase in the early embryo (Fig. 1a–c). The genotypic distribution of the offspring obtained after crossing heterozygous mice was consistent with Mendelian inheritance. PKN1 homozygous knock-in (PKN1 T778A/T778A, hereinafter referred to as “PKN1[T778A]”) mice developed into fertile adults and were morphologically indistinguishable from their wild-type (WT) counterparts (data not shown). Immunoblot analyses of tissue homogenates revealed that PKN1 in PKN1[T778A] mice were approximately 1/4 to 1/2 those in WT mice (Fig. 1d), with some variation among tissues. A real-time PCR analysis of *PKN1* mRNA showed comparable *PKN1* transcript levels in mutant and WT mice (Fig. 1e), suggesting that mutant PKN1 is unstable at the protein level *in vivo*. Immunoprecipitates from the lysate of the PKN1[T778A] mouse spleen, examined using an anti-PKN1 antibody, did not show any autophosphorylation activity, while PKN1 levels were equal in the precipitates from PKN1[T778A] tissues and WT counterparts (Fig. 1f). These results verify that PKN1[T778A] mice completely lacked PKN1 kinase activity, as expected. Therefore, the protein expression of T778A PKN1 may be reduced *in vivo* due to the instability of the unphosphorylated form of PKN1. This hypothesis is supported by a previous analysis indicating that PKN1 expression is reduced in ES cells lacking PDK1, but *PKN1* mRNA exhibits identical expression levels in PDK1 null and PDK1^+/+^ ES cells^26^. The expression levels of other isoforms of PKNs, such as PKN2 and PKN3, were comparable to those of WT counterparts (Fig. 1g).

### PKN1 [T778A] mice exhibit normal lymphoid development, but decreased peripheral blood lymphocytes

**Figure 2:**
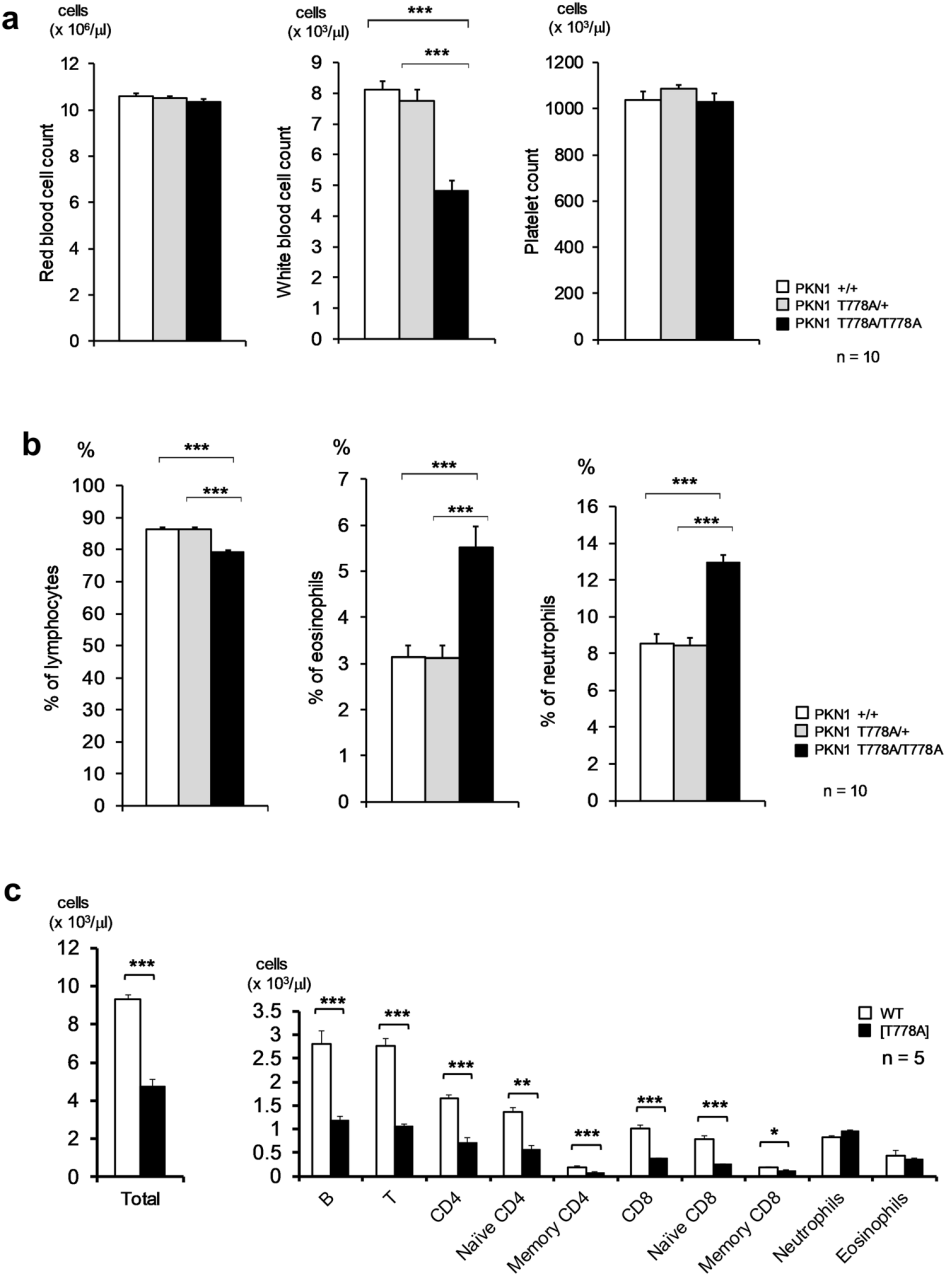
Peripheral blood counts. (**a**) Peripheral blood count. (**b**) Differential white blood cell count. (**c**) Cell population analysis of the peripheral blood. The numbers of total cells and indicated subsets of lymphocytes in the peripheral blood were determined in WT (open bars) and PKN1[T778A] mice (closed bars). Peripheral blood was obtained from 8-week-old mice. Naïve CD4, CD4^+^CD45RB^hi^CD44^lo^; Memory CD4, CD4^+^CD45RB^lo^CD44^hi^; Naïve CD8, CD8^+^CD45RB^hi^CD44^lo^; Memory CD8, CD8^+^CD44^hi^; Neutrophils, SSC^hi^Gr-1^hi^; Eosinophils, SSC^hi^Gr-1^lo^. Data were analysed with unpaired *t*-tests. *P < 0.05, **P < 0.01, ***P < 0.001.

**Figure 3:**
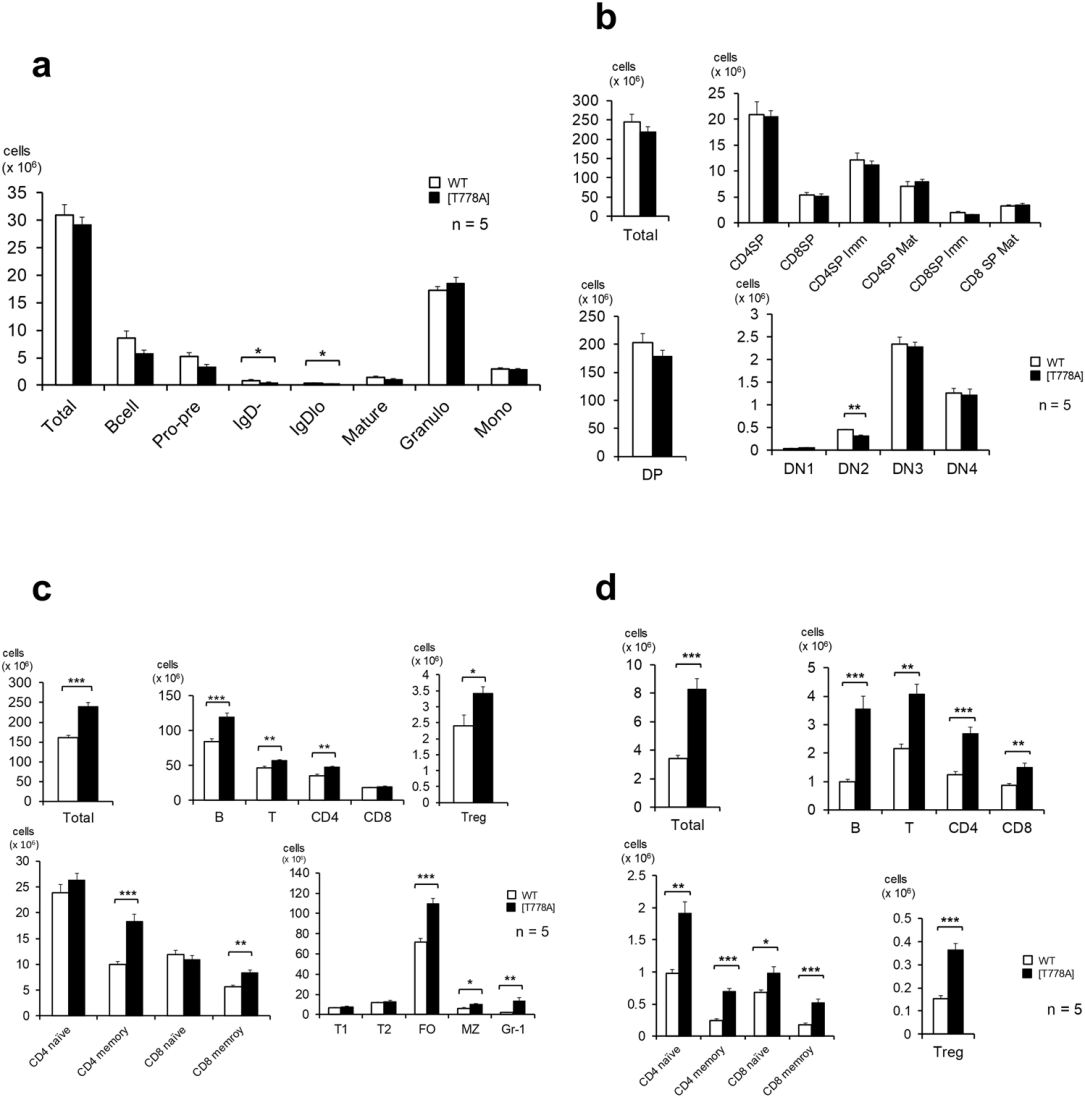
Lymphocyte cellularity of primary and secondary lymphoid organs. Cell population analysis in the bone marrow (**a**), thymus (**b**), spleen (**c**), and lymph nodes (**d**). The numbers of total cells and indicated subsets of white blood cells were determined in 8-week-old WT (open bars) and PKN1[T778A] mice (closed bars). The axillary and inguinal lymph nodes were used as peripheral lymph nodes for analysis. CD4 naïve, CD4^+^CD45RB^hi^CD44^lo^; CD4 memory, CD4^+^CD45RB^lo^CD44^hi^; CD8 naïve, CD8^+^ CD45RB^hi^CD44^lo^; CD8 memory, CD8^+^CD44^hi^; Pro-pre, B220^+^IgM^−^IgD^−^; IgD-, B220^+^IgM^+^IgD^−^; IgD^lo^, B220^+^IgM^+^IgD^lo^; Mature, B220^+^IgM^+^IgD^hi^; Granulo, Gr-1^hi^; Mono, Gr-1^med^CD11b^hi^; CD4SP imm, CD4^+^CD8^−^CD62L^lo^CD69^hi^; CD4SP Mat, CD4^+^CD8^−^CD62L^hi^CD69^lo^; CD8SP Imm, CD4^−^CD8^+^CD62L^lo^CD69^hi^; CD8SP Mat, CD4^−^CD8^+^CD62L^hi^CD69^lo^; DP, CD4^+^CD8^+^; DN1, Lin^−^Thy1.2^+^CD25^−^CD44^+^; DN2, Lin^−^Thy1.2^+^CD25^+^CD44^+^; DN3, Lin^−^Thy1.2^+^CD25^+^CD44^−^; DN4, Lin^−^Thy1.2^+^CD25^−^CD44^−^; Treg, CD4^+^CD25^+^; T1, AA4.1^+^B220^+^IgM^hi^IgD^lo^; T2, AA4.1^+^B220^+^IgM^+^IgD^hi^; FO, B220^+^ IgM^+^CD21^int^CD23^hi^; MZ, B220^+^IgM^+^CD21^hi^CD23^lo^. Data were analysed with unpaired *t*-tests. *P < 0.05, **P < 0.01, ***P < 0.001.

Based on peripheral blood analyses, the white blood cell count was significantly lower in PKN1[T778A] mice than in PKN1 T778A/+ or WT mice, despite comparable red blood cell and platelet counts (Fig. 2a). Differential white blood cell counts revealed that the absolute number of lymphocytes in PKN1[T778A] mice was approximately half that in PKN1 T778A/+ or WT mice, whereas the absolute numbers of eosinophils and neutrophils in PKN1[T778A] mice were comparable to those in WT mice (Fig. 2b and Table 1). Flow cytometric analyses of peripheral circulating white blood cells revealed that both T and B lymphocytes in PKN1[T778A] mice were almost half those in WT mice, and both CD4 (+) and CD8 (+) T cells were significantly reduced in PKN1[T778A] mice (Fig. 2c). Next, T cell development in the thymus and B cell development in the bone marrow were examined based on the expression patterns of cell surface antigens. Flow cytometric analyses of T and B cells in both organs of 8-week-old PKN1[T778A] mice revealed the presence of approximately normal percentages of various thymic, T, and B lineage cell populations; the numbers of cells in these organs were similar to those of WT mice (Fig. 3a and b). Serum level of IgA, IgM, and IgG did not show significant difference between WT and PKN1[T778A] mice, suggesting that the overall normal production of immunoglobulin, without class switch abnormalities in PKN1[T778A] mice (Supplementary Fig. 1). These findings indicate that the T778A mutation in *PKN1* does not have a marked effect on overall lymphocyte development.

**Table 1:** White blood cell cellularity in the peripheral blood (absolute number) (mean ± SEM).

	Lymphocytes (× 10^3^/μl)	Eosinophils (×10^2^/μl)	Neutrophils (×10^2^/μl)
PKN1+/+	8.1 ± 0.3	2.6 ± 0.5	7.1 ± 0.4
PKN1 T778A/+	7.8 ± 0.5	2.5 ± 0.3	6.7 ± 0.4
PKN1 T778A/T778A	4.8 ± 0.2	2.5 ± 0.2	6.1 ± 0.5

### Lymphocytes are sequestered in secondary lymphoid organs

**Figure 4:**
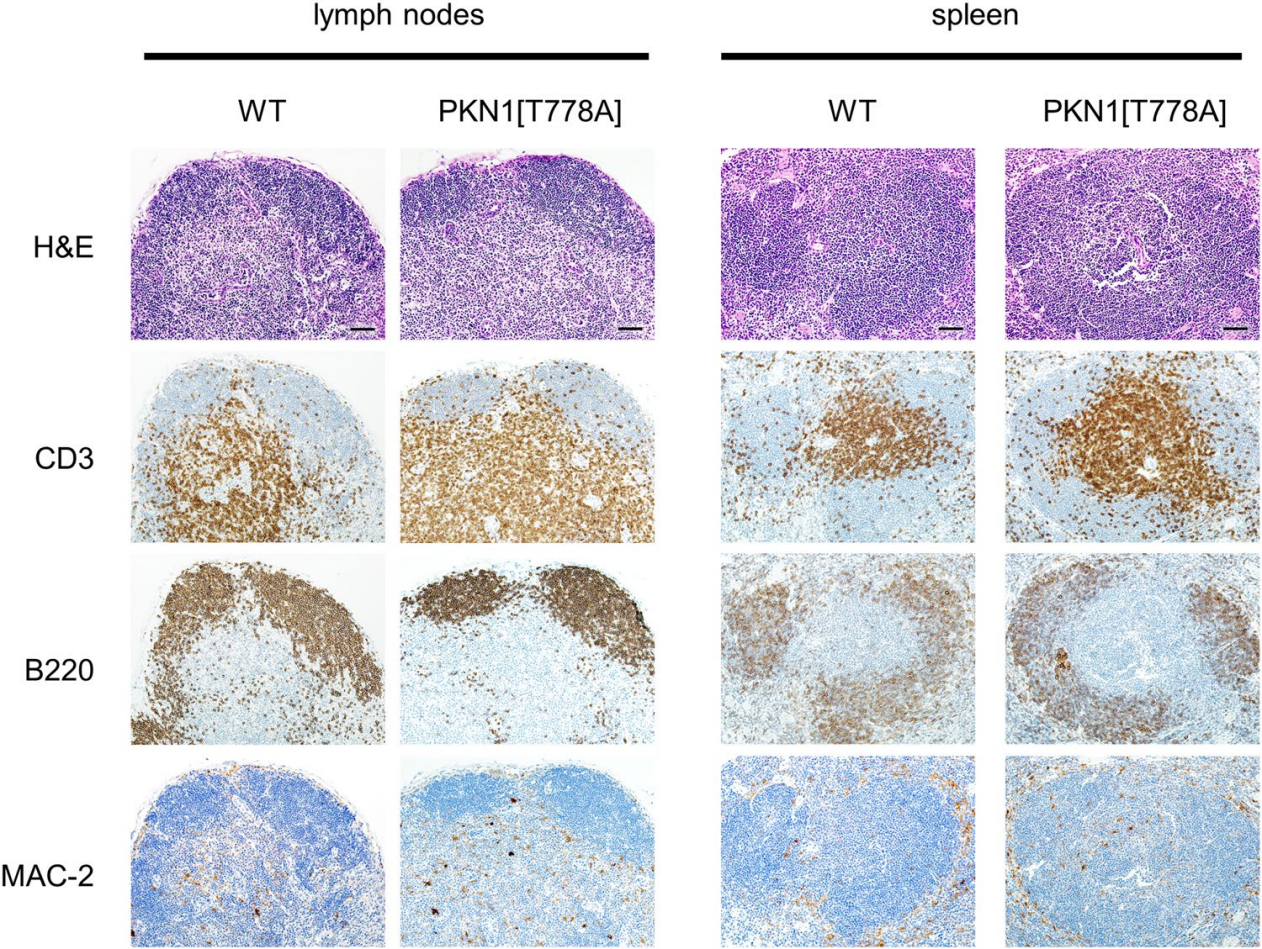
Histological analysis of secondary lymphoid organs. Representative observation from samples of lymph nodes and spleens of 9-week-old mice are shown. H&E, hematoxylin & eosin (H&E) staining; CD3, immunostaining for CD3; B220, immunostaining for B220/CD45R; MAC-2, immunostaining for MAC-2/Galectin-3. Scale bar: 50 μm.

Lymphocyte counts were normal in the thymus and bone marrow and decreased in the peripheral blood; however, the numbers of lymphocytes in the spleen and lymph nodes, i.e., secondary lymphoid organs, in 8-week-old PKN1[T778A] mice were significantly elevated compared with those in WT mice (Fig. 3c and d). There was no significant change in the T-to-B cell ratio, and most lymphocyte subsets accumulated in both the lymph nodes and spleen of PKN1[T778A] mice, suggesting that lymphocytes are generally sequestered to secondary lymphoid organs in PKN1[T778A] mice. However, hematoxylin & eosin staining of lymph node and spleen sections from PKN1[T778A] mice did not show significant structural abnormalities, and immunohistochemical staining revealed similar segregation of T and B cells and macrophage distribution in both organs of PKN1[T778A] mice and WT mice (Fig. 4).

**Figure 5:**
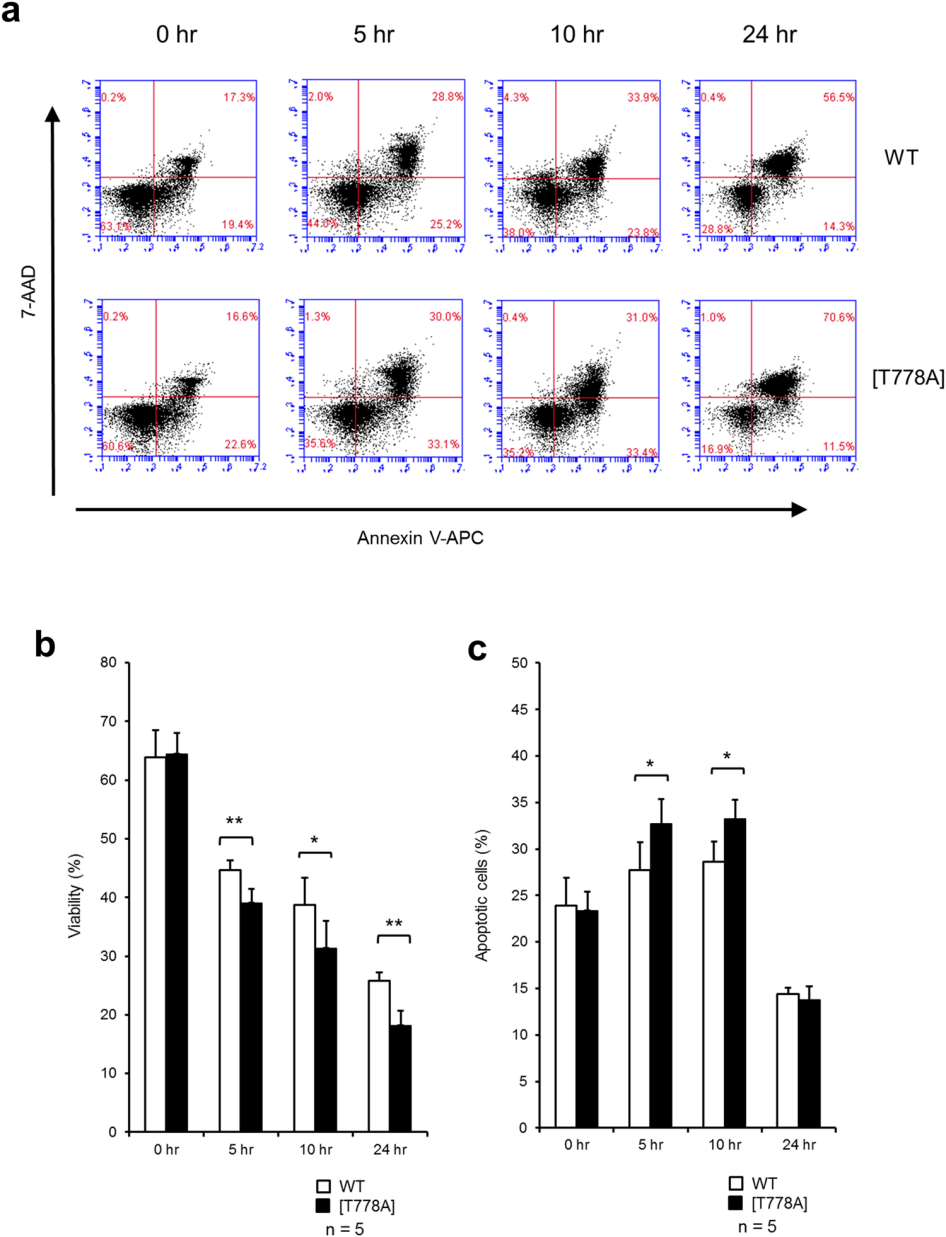
Survival assay of splenic lymphocytes from WT and PKN1[T778A] mice. Viability of lymphocytes were evaluated by flow cytometry with Annexin V-APC and 7-AAD using BD Accuri flow cytometer and Accuri C6 Software. (**a**) Representative dot plots at the indicated time points are shown. (**b**) Viability was evaluated as the percentage of cells that were negative for both Annexin V-APC and 7-AAD. (**c**) The apoptotic cell percentage was quantified from cells that were positive for Annexin V-APC and negative for 7-AAD. Data were analysed with paired t-tests. *P < 0.05. **P < 0.01.

PKN1 has been suggested to inhibit PDK1-mediated activation of PKB/Akt in cells^21, 27^. Since Akt influences cell survival pathways by inhibiting apoptotic processes; the inhibition of PKN1 might promote the overall survival of lymphocytes, leading to their accumulation in secondary lymphoid organs. Therefore, we performed a cell survival assay to examine whether PKN1[T778A] mutant lymphocytes exhibit reduced cell death. Splenic lymphocytes were incubated in complete RPMI 1640 medium containing 10% fetal bovine serum (FBS) for various times and were subjected to flow cytometry using Annexin V-allophycocyanin (APC) and 7-aminoactinomycin D (AAD). As shown in Fig. 5, PKN1[T778A] mutant lymphocytes did not exhibit increased survival, but rather were susceptible to apoptosis and cell death, for as yet unknown reasons. Therefore, the accumulation of lymphocytes in the spleen of PKN1[T778A] mice is not likely due to the anti-apoptotic tendency of lymphocytes.

**Figure 6:**
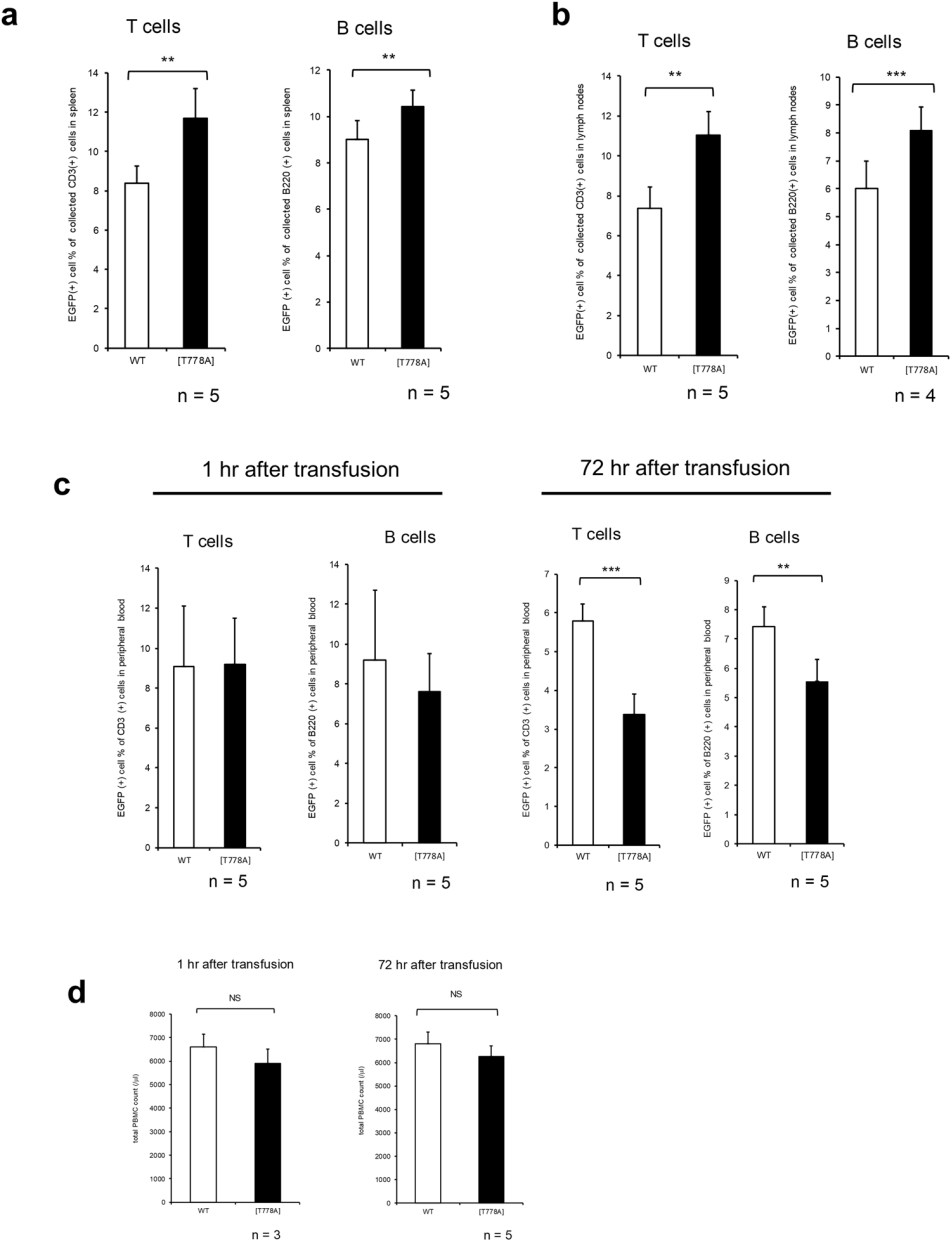
Adoptive transfer experiments. Percentage of EGFP-positive donor lymphocytes collected from the WT recipient spleen (**a**), lymph nodes (**b**), and peripheral blood (**c**). (**d**) Total PBMC count. Peripheral blood was isolated at the indicated time points. Genotypes of donors are labelled at the bottom. Data were analysed with paired *t*-tests. *P < 0.05. **P < 0.01. ***P < 0.001. NS, not significant.

Next, we examined whether the T778A mutation in PKN1 contributes to the aberrant distribution of lymphocytes in a cell-autonomous fashion using an adoptive transfer experiment. To prepare labelled donor lymphocytes for transfusion, enhanced green fluorescent protein gene (*EGFP*) was introduced to both WT and PKN1[T778A] mice by crosses with EGFP transgenic mice. EGFP-labelled splenic lymphocytes were isolated from individual donor mice and were injected into the tail veins of recipient WT mice. The numbers of transferred cells that migrated into the spleen and lymph nodes were determined 7 days after the transfusion. As shown in Fig. 6a and b, significantly more [T778A] mutant than WT T cells and B cells from donor mice were collected from both the spleen and lymph nodes of recipient mice. In contrast, significantly fewer [T778A] mutant than WT T cells and B cells from donor mice were collected from the peripheral blood of recipient mice at 72 hours after transfusion (Fig. 6c), despite no difference in total peripheral blood lymphocytes (Fig. 6d). These results suggest that PKN1[T778A] mutant lymphocytes transfused into WT mice recapitulate the impaired trafficking observed in PKN1[T778A] mice in a cell-autonomous fashion.

### [T778A] mutant lymphocytes exhibit defective migration toward chemokines and sphingosine 1-phosphate (S1P)

**Figure 7:**
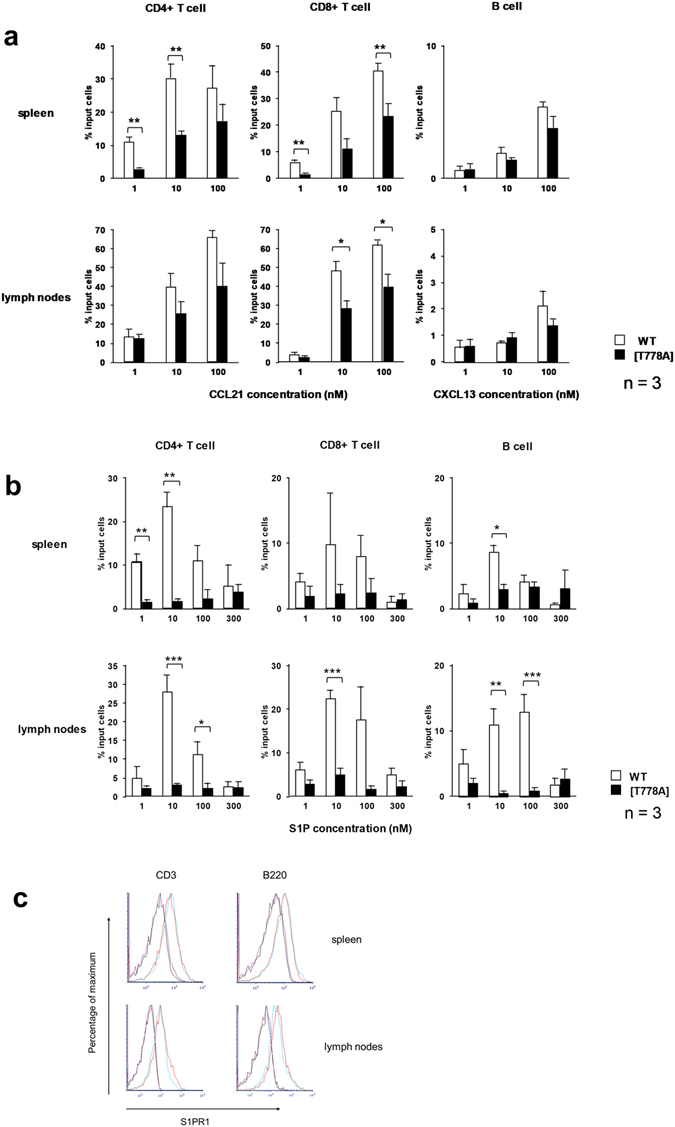
Chemotaxis of lymphocytes *in vitro*. Migration of CD4 (+) T cells, CD8 (+) T cells, and B cells from the spleens and lymph nodes of WT (open bars) and PKN1[T778A] (closed bars) mice toward the indicated chemokines (**a**) and S1P (**b**) was examined using a Transwell chamber. Data were analysed with unpaired *t*-tests. *P < 0.05. **P < 0.01. ***P < 0.001. (**c**) Flow cytometric analysis of the surface expression of S1PR1 on T and B lymphocytes isolated from the spleen and lymph nodes. S1PR1 expression profiles were determined after gating cells based on CD3 or B220/CD45R expression patterns. The profiles shown in black (WT) and violet (PKN1[T778A]) indicate background staining using the isotype control antibody (IgG2A)-APC, and the profiles shown in blue (WT) and red (PKN1[T778A]) indicate staining with the anti S1PR1 antibody-APC.

The lymphocyte distribution is mainly determined by migration to chemokines and the small lipid mediator sphingosine 1-phosphate (S1P)^28^; accordingly, we examined the responses of PKN1 [T778A] lymphocytes to these factors *in vitro*. The chemokine family subset involved in the promotion of the organization and function of secondary lymphoid tissues is referred as homeostatic chemokines, including CC chemokine ligand CCL21 and CXC chemokine ligand CXCL13. These chemokines are important for the entry of lymphocytes into lymph nodes and the white pulp of the spleen. S1P is required for the egress of lymphocytes from secondary lymphoid organs^28, 29^. As shown in Fig. 7a and b, a chemotaxis assay revealed that the migration of [T778A] lymphocytes was mildly impaired toward CCL21 and CXCL13, and was severely impaired toward S1P. The severely impaired egress of PKN1[T778A] lymphocytes from secondary lymphoid organs (compared with the impairment of the entry of lymphocytes into the organs) may explain the PKN1[T778A] mouse phenotype. To examine whether the defective lymphocyte migration in PKN1[T778A] mice is caused at the level of cell surface receptors of S1P, we analysed the expression level of S1PR1, an S1P receptor known to play a major role in the egress of lymphocytes, by flow cytometry. As shown in Fig. 7c, there was no significant difference in the cell surface expression levels of S1PR1 between PKN1[T778A] mutant and WT lymphocytes.

## Discussion

The immune system exhibits a continuous steady state equilibrium of developing cells and effector cells of the lymphoid and myeloid lineages. Although it is estimated that ~70% of the body’s lymphocytes are in lymphoid tissues and 2% are in the blood, lymphocytes equivalent to approximately the entire lymphocyte count in the whole body are trafficked between the blood and lymphoid tissues daily^30, 31^. Newly generated lymphocytes migrate from the bone marrow or thymus into the blood and travel to secondary lymphoid organs, such as the spleen and lymph nodes. Entry from the blood to lymphoid tissues involves a multistep cascade, including selectin-mediated cell rolling, followed by chemokine-triggered integrin activation, integrin-mediated adhesion, and transmigration across the endothelium^32^. After surveying the secondary lymphoid organs, T and B cells egress to the blood and lymph, migrating to other lymphoid organs and continuing surveillance. Egress from the secondary lymphoid organs has not been well-characterized, but some studies have suggested that the major driving force that mediates lymphocyte egress from lymphoid organs is the concentration differential of S1P between luminal and abluminal compartments via stimulation of the lymphocyte S1P receptor^33–36^. The lipid ligand S1P is ubiquitously synthesized, but is largely degraded in most tissues, resulting in low ligand levels in lymphoid tissues and higher concentrations in the blood and lymph^35^. The S1P receptor 1 (S1PR1), one of the five G protein-coupled S1P receptors, has been shown to control lymphocyte egress from the thymus^37, 38^, spleen^37, 39^, and lymph nodes^37^, based on S1PR1 deletion experiments. In this paper, we demonstrated the impaired trafficking of lymphocytes in PKN1[T778A] mice, while PKN1 was not essential for lymphocyte differentiation until at least 8 weeks of age. An *in vitro* chemotaxis analysis and adoptive transfer experiment indicated that the impairment is a PKN1[T778A] mutant lymphocyte cell-autonomous phenotype. PKN1[T778A] lymphocytes showed remarkably less migratory activity toward S1P than that of WT lymphocytes. Therefore, PKN1[T778A] mutant lymphocytes may be less competent to exit from secondary lymphoid organs to the blood and lymph, leading to the accumulation of lymphocytes in secondary lymphoid organs and a decrease in lymphocytes in the peripheral blood of PKN1[T778A] mice. The [T778A] mutant lymphocytes also showed mildly reduced migratory activity toward CCL21 and CXCL13, critical chemokines for the entry of lymphocytes into secondary lymphoid organs; however, predominant suppression of egress from secondary lymphoid organs seems to result in lymphocyte accumulation in these organs. This inference is supported by a previous study indicating that S1PR1 in lymphocytes controls lymph node egress by overcoming retention signals mediated by CCR7 and additional Gαi-coupled receptors^40^. However, it is unlikely that impaired lymphocyte trafficking in PKN1[T778A] mice can be totally attributed to the lack of S1PR1 signalling in lymphocytes, based on the following observations: (i) Although the inhibitory effects of S1PR1 receptor signalling on the egress of lymphocytes from secondary lymphoid organs have been established^37–39^, its effects on recirculating lymphocytes in the spleen are less clear. FTY720 (fingolimod) is a S1P analogue with an antagonistic effect on S1PR1 by inducing proteasome-mediated degradation of S1PR1^41^, creating a pharmacological S1PR1-null state in lymphocytes. The single administration of FTY720 sequesters circulating mature lymphocytes in peripheral lymph nodes and thereby increases the number of lymphocytes in lymph nodes and decreases the number in peripheral blood as well as in the spleen in rats^42^ and mice^43, 44^. However, PKN1[T778A] lymphocytes accumulated in the spleen as well as in lymph nodes (Fig. 3c). (ii) Analyses of S1PR1-deficient fetal liver chimeras^37^ and T cell-specific S1PR1 knockout mice^45^ have revealed peripheral lymphocyte deficiencies and the thymic accumulation of mature CD4 and CD8 single-positive T cells at the same time. This mature thymocyte accumulation has also been observed in mice treated with FTY720^46^. However, PKN1[T778A] mice did not exhibit the accumulation of CD4 and CD8 single-positive T cells in the thymus (Fig. 3b). Other isoforms of S1P receptors might also play roles in impaired lymphocyte trafficking in PKN1[T778A] mice. For instance, S1PR2 may be a candidate, since it has been reported to mediate the confinement of B cells and follicular T helper (Tfh) cells to lymph node germinal centers^47, 48^.

The cell-surface expression level of S1PR1 did not differ significantly between [T778A] and WT lymphocytes (Fig. 7c); therefore, the PKN1[T778A] mutation seems to impair a signalling step downstream of the S1P receptor in lymphocytes. What is the mechanism underlying defective lymphocyte trafficking induced by the PKN1[T778A] mutation? PKN1 has important roles in cell migration and invasion in various adherent cell lines, including prostate adenocarcinoma cell lines, such as PC-3 and LNCaP stimulated by thromboxane and androgen^49–51^, androgen-independent prostate tumour cell lines, such as PC-3M-luc2 and Du145^52^, the triple-negative breast cancer cell line MDA-MB-231^52^, the bladder tumour cell line 5637^53^, and human aortic smooth muscle cells stimulated by monocyte chemotactic protein (MCP)-1^54^. In these cases, PKN1 has been suggested to be involved in processes downstream of Rho or Rac GTPases, major molecular switches of cell migration and cytoskeletal regulation in these cells. Rho and Rac GTPases are also reported to play major roles in lymphocyte migration as follows. (i) RhoA and Rac1 influence chemokine-induced T-cell polarity, which is crucial for *in vivo* migration^55–57^. (ii) Chemokines, such as CCL21, CXCL13, and CXCL12, bind to G-protein-coupled receptors, leading to the activation of Rho and Rac GTPases in B cells^58–60^. (iii) S1P binds to G-protein-coupled S1PR1, which activates the RhoA-specific guanine nucleotide exchange factor (GEF) Lsc (also known as ARHGEF1) and the Rac-specific GEF DOCK2, leading to the migration and egress of lymphocytes from lymph nodes^61, 62^. Indeed, T cells deficient in mDia1, an effector protein of Rho GTPases like PKN1, exhibit impaired egress from the thymus to secondary lymphoid organs, and reduced chemotaxis. Additionally, mDia1 KO mice develop lymphopenia, characterized by diminished T cell populations in lymphoid tissues, but develop without apparent abnormalities and B cell phenotypes, for unknown reasons^63, 64^. In our study, the migration activity of PKN1[T778A] lymphocytes was lower than that of WT cells when stimulated by chemokines (CCL21 for T cells, CXCL13 for B cells, and CXCL12 for T and B cells in Fig. 7a and Supplementary Fig. 2), although the magnitude of the decrease in migration activity was less than that observed in response to S1P stimulation (Fig. 7b). Therefore, it is likely that PKN1 is widely involved in chemokine- and S1P-induced lymphocyte migration downstream of Rho or Rac GTPase functions. PKN1[T778A] mice showed a characteristic set of trafficking abnormalities, i.e. deficiencies in both T and B cells in the peripheral blood, the accumulation of T and B cells in lymph nodes and the spleen, a lack of changes in primary lymphoid organs, and normal numbers of other hematopoietic cells, such as eosinophils, basophils, platelets, and red blood cells. The same set of phenotypes has not been observed in other genetically modified mice to date. Future analyses of PKN1[T778A] mice may unravel novel mechanisms of lymphocyte trafficking. PKN1 has been regarded as promising targets for the treatment of cancers^52, 65, 66^. Therefore, it would be beneficial to clarify the phenotype of PKN1[T778A] mice from the viewpoint of side effects by PKN1 inhibitor for therapeutic purpose.

## Materials and Methods

### Animals

For all studies in this manuscript, mice were compared to littermates or controls, and all were Specific Pathogen Free (SPF). This study was approved by the Institutional Animal Care and Use Committee and carried out according to the Kobe University and Kindai University animal experimentation regulations.

### Generation of PKN1 kinase-negative knock-in mice

A genomic fragment of the mouse *PKN1* gene was isolated from mouse BAC library RPCI-23-394G23 (C57BL/6). The targeting vector for *PKN1* contained a ~8 kbp *Sal*I–*Not*I DNA fragment including exon 10–22 of *PKN1*, pgk-1 promoter-driven neomycin phosphotransferase gene (*pgk-neo*) flanked by two Cre recognition target (loxP) sites, and a ~4 kbp *Swa*I/*Xho*I DNA fragment and followed by the diphtheria toxin (DT) gene for negative selection (Fig. 1a). The kinase-negative knock-in vector was constructed by introducing this targeting vector with the point mutation encoding alanine instead of threonine-778 and insertion of oligonucleotide encoding FLAG peptide to the 3′-terminal of the coding region of *PKN1* in exon 22 (Fig. 1). We used the C57BL/6-derived ES cell line RENKA^67^ for the gene targeting. The ES cells were cultured as described^68^. The targeting vector was linearized and electroporated into ES cells by using Gene Pulser Xcell (Bio-Rad, Hercules, CA). G-418 selection (175 μg/ml) was started 36–48 hours after electroporation and continued for 1 week. The recombinant ES cell clones were identified by Southern blot analyses, and were injected into 8-cell-stage embryos of ICR mice. The embryos were cultured to blastocysts and transferred to the uterus of pseudopregnant ICR mice. The resultant chimeric mice were mated with C57BL/6 mice and the F1 mice were screened by Southern blot to establish the mutant PKN1 F1 mouse line. The mutant F1 mice were crossed with the transgenic strain expressing a Cre recombinase gene under the direction of EIIα promoter^25^ to remove neomycin resistant gene (neo) cassette and later isolated PKN1 mutant strain lacking Cre gene, and were backcrossed at least 10 times into the Charles River C57BL/6N background before phenotypic analysis. The resultant PKN1[T778A] mutant mice were born at a frequency expected for Mendelian inheritance.

### Genotyping

Genomic DNA was isolated from ES cells and mouse tail snips by standard techniques and subjected to Southern blot analysis and PCR analysis for identification. Southern blot analysis was performed using genomic DNA digested with *Nhe*I/*Not*I and *Bam*HI/*Hin*dIII probed with probe A and probe B, respectively, as indicated in the Fig. 1a. Wild type (“wt” in Fig. 1a) and mutant (“mt” in Fig. 1a) alleles containing Neo cassettes are indicated by the presence of a 15 kbp versus 11.5 kbp DNA fragment for probing with probe A, and a 12.5 kbp versus 5 kbp DNA fragment for probing with probe B (Fig. 1b). Genotyping of mouse tail, for discrimination of wt and mutant lacking Neo cassette (“ki” in Fig. 1a) was performed using the PCR primers N1-geF (5′-GCCTCTGTGTGCATCTGG–3′), N1-geR (5′-CCTTCTACCCACACGGCC-3′), yielding PCR products of 170 bp (wt) and 270 bp (ki) (Fig. 1c). Reaction conditions were as follows: 96 °C for 5 min for 1 cycle, and 96 °C for 1 min, 63 °C for 1 min, 72 °C for 1 min for 35 cycles, and 72 °C for 5 min for 1 cycle.

### RT-qPCR (Reverse transcription quantitative polymerase chain reaction)

Total RNA of mouse thymus, spleen and lymph node were isolated by using an RNeasy mini kit (QIAGEN), according to the manufacturer’s instructions. The cDNAs were synthesized using ReverTra Ace qPCR RT Master Mix (TOYOBO) according to the manufacturer’s protocols. qPCR was performed (45 cycles of 10 seconds at 95 °C, 10 seconds at 60 °C, and 10 seconds at 72 °C) with Thunderbird SYBR qPCR Mix (TOYOBO) using the LightCycler480 System (Roche). The sense primer for *PKN1* was (5′-TGCTCTATGAGATGTTGGTTGGA-3′) and the antisense primer was 5′-CAGACAGGAAGCGGGGATAG-3′). Expression of *GAPDH* was used as an endogenous control. ΔΔCt method was used to determine relative fold expression of mRNA.

### Antibodies

The polyclonal antibodies αN2 and αC6 for PKN1 were prepared as described^69^. The polyclonal antibody αNUS for detection of PKN3 was prepared as described^70^. Anti PRK2 antibody for PKN2 was purchased from BD Transduction Laboratories, and anti phospho-PRK1 (Thr774)/PRK2 (Thr816) antibody was from Cell Signaling Technology. Annexin V-APC and 7-ADD were purchased from BD Biosciences. The mAbs used for flow cytometric analyses for cell surface antigens were purchased from BD Biosciences, eBioscience or BioLegend. Anti-mouse EDG-1/S1PR1-APC MAb (Clone 713412) and Rat IgG2A-APC Isotype Control were purchased from R&D Systems and used for S1PR1 receptor assay. Anti CD3 rabbit monoclonal antibody (clone SP7; Abcam), an anti B220/CD45R rat monoclonal antibody (clone RA3-6B2; Abcam) and an anti MAC-2/Galectin-3 rat monoclonal antibody (clone M3/38; Cedarlane) were used for immunohistochemical analysis.

### Immunoblot analysis

Mouse organs and cells were lysed in SDS-sample buffer, boiled, and were subjected to 6–10% SDS-PAGE and separated products were subsequently transferred to a polyvinylidene difluoride membrane. The membrane was then blocked with TBS (20 mM Tris/HCl at pH 7.5, 137 mM NaCl) containing 0.05% Triton X-100 (TBS-T) and 5% normal goat serum or Blocking One (nacalai tesque) for 1 hour at room temperature. The membrane was then incubated in TBS-T and the primary antibody for 1 hour at room temperature or for O/N at 4 °C. The membrane was washed three times (5 min each time) in TBS-T before incubating the blot in TBS-T containing the secondary antibody conjugated to horseradish peroxidase at 1:2000–1:10,000 dilution for 45 min. After this incubation, the membrane was subjected to three 10 min washes in TBS-T. Blots were developed by the enhanced chemiluminescence method.

### Immunoprecipitation and kinase assay

Spleens were isolated from WT and PKN1[T778A] mice and homogenized in homogenate buffer (50 mM Tris/HCl at pH 7.5, 150 mM NaCl, 1 mM EDTA, 3 mM MgCl_2_, 1 mM DTT, 1 mM PMSF, 5 μg/ml leupeptin, 1 mM NaF, 1% NP-40). After centrifugation at 13,000 x g for 30 min, cleared lysates were incubated with αN2 at 4 °C for 2 hours, and the protein A-Sepharose (GE Healthcare) was added and incubated for further 30 min. The resulting immunoprecipitates were subjected to *in vitro* kinase assay and immunoblot analysis using αC6. For autophosphorylation reaction, the immunoprecipitates were incubated in 25 μl reaction mixture (20 mM Tris–HCl, pH 7.5, 4 mM MgCl_2_, 100 μM ATP, 18.5 KBq [γ-^32^P]ATP) at 30 °C. After 5 min, the reaction was stopped by addition of SDS sample buffer and boiled for 5 min. A 20 μl aliquot of reaction mixture was subjected to SDS–PAGE. The gel was fixed, dried, and subjected to autoradiography using BAS2000 imaging analyzer (Fuji film).

### Peripheral blood count

Peripheral blood of mice was collected from the retro-orbital venous plexus, and measured by an automatic hamatology analyzer (ADVIA120, SIEMENS) and also manually using hemocytometer for Fig. 2 and Table 1.

### Flow cytometry

Cell suspensions were prepared from bone marrow and spleen were treated with 1 x ACK buffer (150 mM NH_4_Cl, 10 mM KHCO_3_, 1 mM EDTA) to remove red blood cells, washed, and incubated with anti CD16/CD32 (BD Fc block) to block binding of conjugated antibodies to FcγR. Cell suspensions from thymus and lymph nodes were directly incubated with anti CD16/CD32 antibody.

After cells were labeled with appropriate primary antibodies for 30 min on ice and fixed in 1% paraformaldehyde in PBS. They were subjected to flow cytometry using FACSCalibur or Accuri (both from BD Biosciences) with FlowJo (FlowJo, LLC) or FCS Express 6 (De Novo Software) to determine the fraction of each subset. The surface expression of S1PR1 was evaluated using flow cytometry with anti-S1PR1-APC antibody. Staining with S1PR1 antibody was performed in the presence of fatty acid–free BSA (Sigma-Aldrich).

### *In vitro* cell survival assay

Spleens were dissected out after decapitation of 7- to 9-week-old mice under anesthesia and shedding of circulating blood. Erythrocytes were lysed by 1 x ACK buffer, and isolated lymphocytes were suspended in MACS buffer (PBS containing 0.5% fatty acid-free BSA and 2 mM EDTA). One million of isolated splenic lymphocytes were incubated with RPMI 1640 containing 2 mM glutamine, 1% Penicillin G/Streptomycin, and 10% fetal bovine serum in a 3.5 cm dish at 37 °C in 5% CO_2_ chamber. After 5, 10, and 24 hours, cells were washed and incubated with 7-AAD and Annexin V-APC for 15 min at room temperature. Cells were subjected to flow cytometry (BD Accuri), and those found positive for Annexin V and negative for 7-AAD were quantified as “apoptotic cells”, and those found negative for both Annexin V and 7-AAD were quantified as “viable cells”.

### Adoptive transfer

Transgenic mouse strain expressing EGFP under the control of CAG promoter was provided from Kumamoto university (CARD ID 2045). EGFP gene was introduced into WT and PKN1[T778A] mice by intercrossing of these mice with the EGFP transgenic mice. After anesthesia, 7- to 9-week-old EGFP and EGFP;PKN1[T778A] mice were decapitated and spleens were dissected out, followed by isolation of splenic lymphocytes with the aid of 1 x ACK buffer and MACS buffer as described in “*in vitro* cell survival assay” section. Isolated donor lymphocytes from EGFP and EGFP;PKN1[T778A] mice were suspended in Hank’s Balanced Salt Solution (HBSS), and 5 × 10^7^ lymphocytes from each mouse were transfused into individual WT recipient mouse through tail vein, respectively. One hour after transfusion, ~50 μl of blood was collected from tail vein of each recipient mice. Peripheral Blood Mono nuclear Cells (PBMCs) were quantified using Turk’s solution, which was followed by flow cytometry (BD Accuri) with anti CD3-APC (for T lymphocyte) and anti B220/CD45R-APC (for B lymphocyte) antibodies. After gating through FL4 filter for APC, the percentage of EGFP-positive lymphocyte population was quantified using FL1 filter in recipient mice. Seventy-two hours after transfusion the same procedures were repeated. On the 7th day, recipient WT mice were sacrificed, and spleen and inguinal lymph nodes were dissected out, followed by lymphocyte isolation. Isolated lymphocytes were subjected to flow cytometry as described above to quantify the percentage of EGFP-positive lymphocyte population.

### Histological analysis

Spleen and lymph nodes from 9-week-old mice were fixed in 4% paraformaldehyde and embedded in paraffin wax for histological analysis. Three-μm sections were processed after deparaffinization. The sections were stained with hematoxylin and eosin solution. Immunostaining for CD3, B220/CD45R and MAC-2/Galectin-3 was performed using the Universal Immunoenzyme Polymer method (Nichirei Bioscience) with an anti-CD3 rabbit monoclonal antibody, an anti-B220/CD45R rat monoclonal antibody, and an anti MAC-2/Galectin-3 rat monoclonal antibody, respectively. The signal was developed with a diaminobenzidine solution (Dako). HE-stained sections and immunostained sections were recorded by a BX-51 light microscope (Olympus).

### *In vitro* migration assay

CCL21 and CXCL13 were purchased from R&D Systems. S1P was purchased from Sigma. *In vitro* migration assay was conducted using 96-well chemoTx chamber with 5-μm pore inserts (Neuroprobe) as described previously^71^. In brief, single cells prepared from mouse spleen or lymph nodes were suspended at 8 × 10^6^/ml in RPMI 1640 containing 1 mg/ml BSA and 20 mM HEPES (pH 7.4), and applied to upper wells (25 μl/well). The same medium without or with CCL21, CXCL13, or S1P at indicated concentrations was applied to lower wells (29 μl/well). After 1 h at 37 °C, the content of each lower well was transferred to a polypropylene pointed-bottom tube. The cells were pelleted by centrifugation at 200 × g for 5 min, resuspended in 0.1% BSA in PBS, and stained with APC-Cy7-labeled anti-mouse CD4, PerCP-Cy5.5-labeled anti-mouse CD8, and FITC-labeled anti-mouse CD19. After washing, cells were analyzed on a FACSFortessa (BD Biosciences) and analyzed with FlowJo (FlowJo, LLC). All assays were done in duplicate.

### Statistical analysis

All experiments were performed independently in at least triplicate and a statistical significance was calculated by Student’s unpaired *t*-test and Student’s paired *t*-test to examine the differences between the two groups of data. A p value < 0.05 was considered significant. Data displayed in the figures and text represent mean ± standard error (SEM) of representative experiments unless otherwise stated.

### Data availability

The datasets generated during and/or analysed during the current study are available from the corresponding author on reasonable request.

## Electronic supplementary material

Supplementary Information

## References

[1] Mukai H (2003). The structure and function of PKN, a protein kinase having a catalytic domain homologous to that of PKC. J Biochem..

[2] Maesaki R (1999). The structural basis of Rho effector recognition revealed by the crystal structure of human RhoA complexed with the effector domain of PKN/PRK1. Mol Cell..

[3] Mukai H (1994). Activation of PKN, a novel 120-kDa protein kinase with leucine zipper-like sequences, by unsaturated fatty acids and by limited proteolysis. Biochem Biophys Res Commun..

[4] Peng B, Morrice NA, Groenen LC, Wettenhall RE (1996). Phosphorylation events associated with different states of activation of a hepatic cardiolipin/protease-activated protein kinase. Structural identity to the protein kinase N-type protein kinases. J Biol Chem..

[5] Amano M (1996). Identification of a putative target for Rho as the serine-threonine kinase protein kinase N. Science..

[6] Watanabe G (1996). Protein kinase N (PKN) and PKN-related protein rhophilin as targets of small GTPase Rho. Science..

[7] Shibata H (1996). Characterization of the interaction between RhoA and the amino-terminal region of PKN. FEBS Lett..

[8] Hutchinson CL, Lowe PN, McLaughlin SH, Mott HR, Owen D (2013). Differential binding of RhoA, RhoB, and RhoC to protein kinase C-related kinase (PRK) isoforms PRK1, PRK2, and PRK3: PRKs have the highest affinity for RhoB. Biochemistry..

[9] Torbett NE, Casamassima A, Parker PJ (2003). Hyperosmotic-induced protein kinase N 1 activation in a vesicular compartment is dependent upon Rac1 and 3-phosphoinositide-dependent kinase 1. J Biol Chem..

[10] Owen D (2003). Molecular dissection of the interaction between the small G proteins Rac1 and RhoA and protein kinase C-related kinase 1 (PRK1). J Biol Chem..

[11] Flynn P, Mellor H, Palmer R, Panayotou G, Parker PJ (1998). Multiple interactions of PRK1 with RhoA. Functional assignment of the Hr1 repeat motif. J Biol Chem..

[12] Mukai H (1997). Interaction of PKN with alpha-actinin. J Biol Chem..

[13] Vincent S, Settleman J (1997). The PRK2 kinase is a potential effector target of both Rho and Rac GTPases and regulates actin cytoskeletal organization. Mol Cell Biol..

[14] Calautti E (2002). Fyn tyrosine kinase is a downstream mediator of Rho/PRK2 function in keratinocyte cell-cell adhesion. J Cell Biol..

[15] Wallace SW, Magalhaes A, Hall A (2011). The Rho target PRK2 regulates apical junction formation in human bronchial epithelial cells. Mol Cell Biol..

[16] Isagawa T, Takahashi M, Kato T, Mukai H, Ono Y (2005). Involvement of protein kinase PKN1 in G2/M delay caused by arsenite. Mol Carcinog..

[17] Schmidt A, Durgan J, Magalhaes A, Hall A (2007). Rho GTPases regulate PRK2/PKN2 to control entry into mitosis and exit from cytokinesis. EMBO J..

[18] Misaki K (2001). PKN delays mitotic timing by inhibition of Cdc25C: possible involvement of PKN in the regulation of cell division. Proc Natl Acad Sci USA.

[19] Metzger E, Muller JM, Ferrari S, Buettner R, Schule R (2003). A novel inducible transactivation domain in the androgen receptor: implications for PRK in prostate cancer. EMBO J..

[20] Leenders F (2004). PKN3 is required for malignant prostate cell growth downstream of activated PI 3-kinase. EMBO J..

[21] Yasui T (2012). Protein kinase N1, a cell inhibitor of Akt kinase, has a central role in quality control of germinal center formation. Proc Natl Acad Sci USA.

[22] Oishi K (2001). PKN regulates phospholipase D1 through direct interaction. J Biol Chem..

[23] Takahashi M (2003). Regulation of a mitogen-activated protein kinase kinase kinase, MLTK by PKN. J Biochem..

[24] Yoshinaga C, Mukai H, Toshimori M, Miyamoto M, Ono Y (1999). Mutational analysis of the regulatory mechanism of PKN: the regulatory region of PKN contains an arachidonic acid-sensitive autoinhibitory domain. J Biochem..

[25] Lakso M (1996). Efficient *in vivo* manipulation of mouse genomic sequences at the zygote stage. Proc Natl Acad Sci USA.

[26] Balendran A, Hare GR, Kieloch A, Williams MR, Alessi DR (2000). Further evidence that 3-phosphoinositide-dependent protein kinase-1 (PDK1) is required for the stability and phosphorylation of protein kinase C (PKC) isoforms. FEBS Lett..

[27] Wick MJ, Dong LQ, Riojas RA, Ramos FJ, Liu F (2000). Mechanism of phosphorylation of protein kinase B/Akt by a constitutively active 3-phosphoinositide-dependent protein kinase-1. J Biol Chem..

[28] Cyster JG (2005). Chemokines, sphingosine-1-phosphate, and cell migration in secondary lymphoid organs. Annual review of immunology..

[29] Stein JV, Nombela-Arrieta C (2005). Chemokine control of lymphocyte trafficking: a general overview. Immunology..

[30] Trepel, F. Number and Distribution of Lymphocytes in Man. A Critical Analysis. *Klin Wschr* (1974). 10.1007/BF01468720 4853392

[31] Pabst, J. W. A. R. Distribution of lymphocyte subsets and natural killer cells in the human body. *Clin Investig* (1992). 10.1007/BF00184787 1392422

[32] von Andrian UH, Mempel TR (2003). Homing and cellular traffic in lymph nodes. Nature reviews Immunology..

[33] Simmons S, Ishii M (2014). Sphingosine-1-phosphate: a master regulator of lymphocyte egress and immunity. Arch Immunol Ther Exp (Warsz)..

[34] Lo CG, Xu Y, Proia RL, Cyster JG (2005). Cyclical modulation of sphingosine-1-phosphate receptor 1 surface expression during lymphocyte recirculation and relationship to lymphoid organ transit. J Exp Med..

[35] Schwab SR (2005). Lymphocyte sequestration through S1P lyase inhibition and disruption of S1P gradients. Science..

[36] Pappu R (2007). Promotion of lymphocyte egress into blood and lymph by distinct sources of sphingosine-1-phosphate. Science..

[37] Matloubian M (2004). Lymphocyte egress from thymus and peripheral lymphoid organs is dependent on S1P receptor 1. Nature..

[38] Allende ML (2008). S1P1 receptor expression regulates emergence of NKT cells in peripheral tissues. Faseb j..

[39] Cinamon G (2004). Sphingosine 1-phosphate receptor 1 promotes B cell localization in the splenic marginal zone. Nat Immunol..

[40] Pham TH, Okada T, Matloubian M, Lo CG, Cyster JG (2008). S1P1 receptor signaling overrides retention mediated by G alpha i-coupled receptors to promote T cell egress. Immunity..

[41] Oo ML (2007). Immunosuppressive and anti-angiogenic sphingosine 1-phosphate receptor-1 agonists induce ubiquitinylation and proteasomal degradation of the receptor. J Biol Chem..

[42] Chiba K (1998). FTY720, a novel immunosuppressant, induces sequestration of circulating mature lymphocytes by acceleration of lymphocyte homing in rats. I. FTY720 selectively decreases the number of circulating mature lymphocytes by acceleration of lymphocyte homing. J Immunol..

[43] Mandala S (2002). Alteration of lymphocyte trafficking by sphingosine-1-phosphate receptor agonists. Science..

[44] Luo ZJ, Tanaka T, Kimura F, Miyasaka M (1999). Analysis of the mode of action of a novel immunosuppressant FTY720 in mice. Immunopharmacology..

[45] Allende ML, Dreier JL, Mandala S, Proia RL (2004). Expression of the sphingosine 1-phosphate receptor, S1P1, on T-cells controls thymic emigration. J Biol Chem..

[46] Yagi H (2000). Immunosuppressant FTY720 inhibits thymocyte emigration. Eur J Immunol..

[47] Green JA (2011). The sphingosine 1-phosphate receptor S1P(2) maintains the homeostasis of germinal center B cells and promotes niche confinement. Nat Immunol..

[48] Moriyama S (2014). Sphingosine-1-phosphate receptor 2 is critical for follicular helper T cell retention in germinal centers. J Exp Med..

[49] O’Sullivan, A. G., Mulvaney, E. P., Hyland, P. B. & Kinsella, B. T. Protein kinase C-related kinase 1 and 2 play an essential role in thromboxane-mediated neoplastic responses in prostate cancer. *Oncotarget* (2015). 10.18632/oncotarget.4664 PMC4694913 26296974

[50] O’Sullivan, A. G., Mulvaney, E. P. & Kinsella, B. T. Regulation of Protein Kinase C-related Kinase (PRK) signalling by the TPα and TPβ isoforms of the human Thromboxane A2 receptor: Implications for Thromboxane- and Androgen- dependent Neoplastic and Epigenetic Responses in Prostate Cancer. *Biochimica et Biophysica Acta* (*BBA*) *- Molecular Basis of Disease* (2017). 10.1016/j.bbadis.2017.01.011 28108419

[51] Turner, E. C. *et al*. Identification of an interaction between the TP&alpha; and TP{beta} isoforms of the human thromboxane A2 receptor with protein kinase C-related kinase (PRK) 1. Implications for prostate cancer. *J Biol Chem* (2011). 10.1074/jbc.M110.181180 PMC3083147 21357687

[52] Jilg, C. A. *et al*. PRK1/PKN1 controls migration and metastasis of androgen-independent prostate cancer cells. *Oncotarget* (2014). 10.18632/oncotarget.2653 PMC4350344 25504435

[53] Lachmann S (2011). Regulatory domain selectivity in the cell-type specific PKN-dependence of cell migration. PLoS One..

[54] Singh, N. K. *et al*. Protein Kinase N1 Is a Novel Substrate of NFATc1-mediated Cyclin D1-CDK6 Activity and Modulates Vascular Smooth Muscle Cell Division and Migration Leading to Inward Blood Vessel Wall Remodeling. *J Biol Chem* (2012). 10.1074/jbc.M112.361220 PMC3476296 22893700

[55] Stowers L, Yelon D, Berg LJ, Chant J (1995). Regulation of the polarization of T cells toward antigen-presenting cells by Ras-related GTPase CDC42. Proc Natl Acad Sci USA.

[56] Sanchez-Madrid F, del Pozo MA (1999). Leukocyte polarization in cell migration and immune interactions. EMBO J..

[57] del Pozo MA, Vicente-Manzanares M, Tejedor R, Serrador JM, Sanchez-Madrid F (1999). Rho GTPases control migration and polarization of adhesion molecules and cytoskeletal ERM components in T lymphocytes. Eur J Immunol..

[58] Henderson RB (2010). A novel Rac-dependent checkpoint in B cell development controls entry into the splenic white pulp and cell survival. J Exp Med..

[59] Fukui Y (2001). Haematopoietic cell-specific CDM family protein DOCK2 is essential for lymphocyte migration. Nature..

[60] Montresor A (2009). Comparative analysis of normal versus CLL B-lymphocytes reveals patient-specific variability in signaling mechanisms controlling LFA-1 activation by chemokines. Cancer Res..

[61] Rubtsov A (2005). Lsc regulates marginal-zone B cell migration and adhesion and is required for the IgM T-dependent antibody response. Immunity..

[62] Nombela-Arrieta C (2007). A central role for DOCK2 during interstitial lymphocyte motility and sphingosine-1-phosphate-mediated egress. J Exp Med..

[63] Eisenmann KM (2007). T cell responses in mammalian diaphanous-related formin mDia1 knock-out mice. J Biol Chem..

[64] Sakata D (2007). Impaired T lymphocyte trafficking in mice deficient in an actin-nucleating protein, mDia1. J Exp Med..

[65] Kohler J (2012). Lestaurtinib inhibits histone phosphorylation and androgen-dependent gene expression in prostate cancer cells. PLoS One..

[66] Metzger E (2008). Phosphorylation of histone H3 at threonine 11 establishes a novel chromatin mark for transcriptional regulation. Nat Cell Biol..

[67] Fukaya M (2006). Abundant distribution of TARP gamma-8 in synaptic and extrasynaptic surface of hippocampal neurons and its major role in AMPA receptor expression on spines and dendrites. Eur J Neurosci..

[68] Mishina M, Sakimura K (2007). Conditional gene targeting on the pure C57BL/6 genetic background. Neurosci Res..

[69] Mukai H (1996). Translocation of PKN from the cytosol to the nucleus induced by stresses. Proc Natl Acad Sci USA.

[70] Mukai H (2016). PKN3 is the major regulator of angiogenesis and tumor metastasis in mice. Sci Rep..

[71] Matsuo K (2016). CCR4 is critically involved in effective antitumor immunity in mice bearing intradermal B16 melanoma. Cancer Lett..

