# Supplementary material for: Impaired lymphocyte trafficking in mice deficient in the kinase activity of PKN1
Source: Sci Rep. 2017 Aug 9;7:7663. doi: 10.1038/s41598-017-07936-9 (PMC5550459; doi:10.1038/s41598-017-07936-9)

Supplementary Information

Impaired lymphocyte trafficking in mice deficient in the kinase activity of PKN1

Rana Mashud[1], Akira Nomachi[2], Akihide Hayakawa[3], Koji Kubouchi[1], Sally Danno[1], Takako Hirata[4], Kazuhiko Matsuo[5], Takashi Nakayama[5], Ryosuke Satoh[6], Reiko Sugiura[6], Manabu Abe[7], Kenji Sakimura[7], Shigeharu Wakana[8], Hiroyuki Ohsaki[9], Shingo Kamoshida[9], and Hideyuki Mukai[1,10*]

[1]Graduate School of Medicine, Kobe University, Kobe 657-8501, Japan

[2]Center for Innovation in Immunoregulative Technology and Therapeutics, Kyoto University Graduate School of Medicine, Kyoto, Japan

[3]Graduate School of Science and Technology, Kobe University, Kobe 657-8501, Japan

[4]Department of Fundamental Biosciences, Shiga University of Medical Science, Seta-Tsukinowa-cho Otsu, Shiga 520-2192, Japan

[5]Division of Chemotherapy, Kindai University School of Pharmacy, Kowakae, Higashi-Osaka 577-8502, Japan

[6]Laboratory of Molecular Pharmacogenomics, School of Pharmaceutical Sciences, Kindai University, 3-4-1 Kowakae, Higashi-Osaka 577-8502, Japan

[7]Department of Cellular Neurobiology, Brain Research Institute, Niigata University, Niigata 951-8585, Japan

[8]Japan Mouse Clinic, RIKEN BioResource Center, 3-1-1 Koyadai,Tsukuba-shi, Ibaraki 305-0074, Japan

[9]Laboratory of Pathology, Department of Medical Biophysics, Kobe University Graduate School of Health Sciences, 7-10-2 Tomogaoka, Suma, Kobe, Hyogo 654-0142, Japan

[10]Biosignal Research Center, Kobe University, Kobe 657-8501, Japan

* to whom correspondence should be addressed (mukinase@kobe-u.ac.jp).

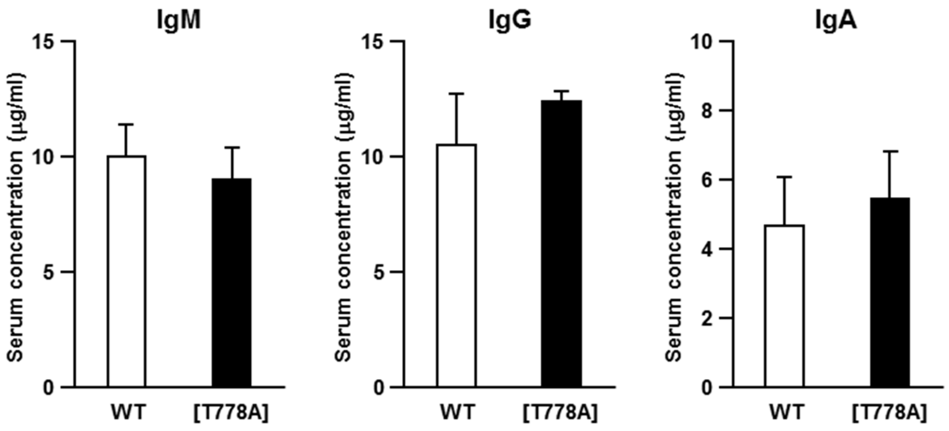

Supplementary Fig. 1. Total IgM, IgG, and IgA levels in serum.

Total IgM, IgG and IgA concentration in serum were measured using the mouse IgM, IgG or IgA ELISA kits (Thermo Fisher Scientific Inc.) following the manufacturer's instructions. Serum samples were obtained from WT and PKN1[T778A] mice. Total IgM, IgG, and IgA concentrations were quantified by ELISA. The data are expressed as mean ± SE of results from 5 mice.

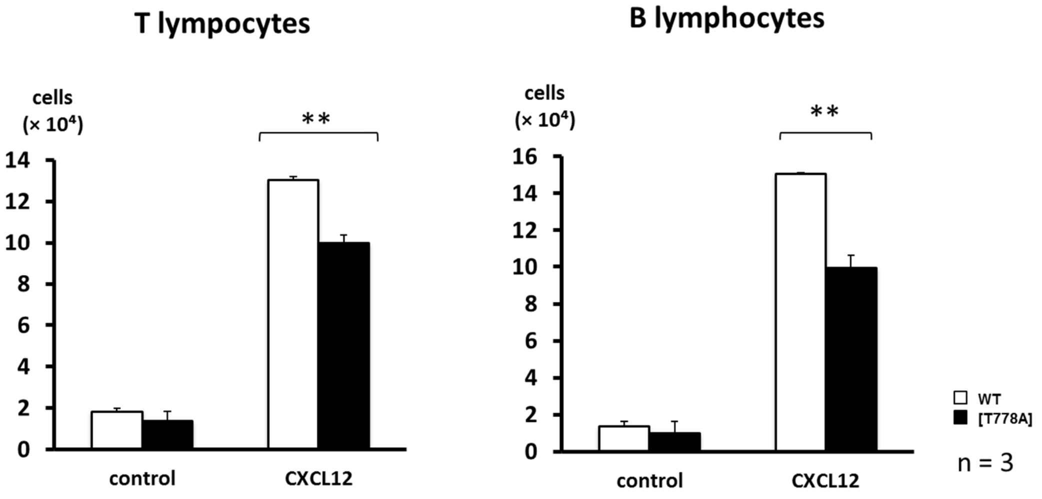

Supplementary Fig. 2. Chemotaxis toward CXCL12 *in vitro*.

T cells and B cells were pre-isolated from WT and PKN1[T778A] mouse spleens by using mouse Pan T Cell Isolation Kit II (Miltenyi) and mouse B Cell Isolation Kit (Miltenyi) for T cells and B cells, respectively, and were subjected to transwell migration assay in the presence or absence of 200 ng/ml CXCL12 as indicated. Data were analyzed with unpaired t-test. ** indicates P<0.01.

Supplementary Fig. 3. Uncropped scans

Figure 1b

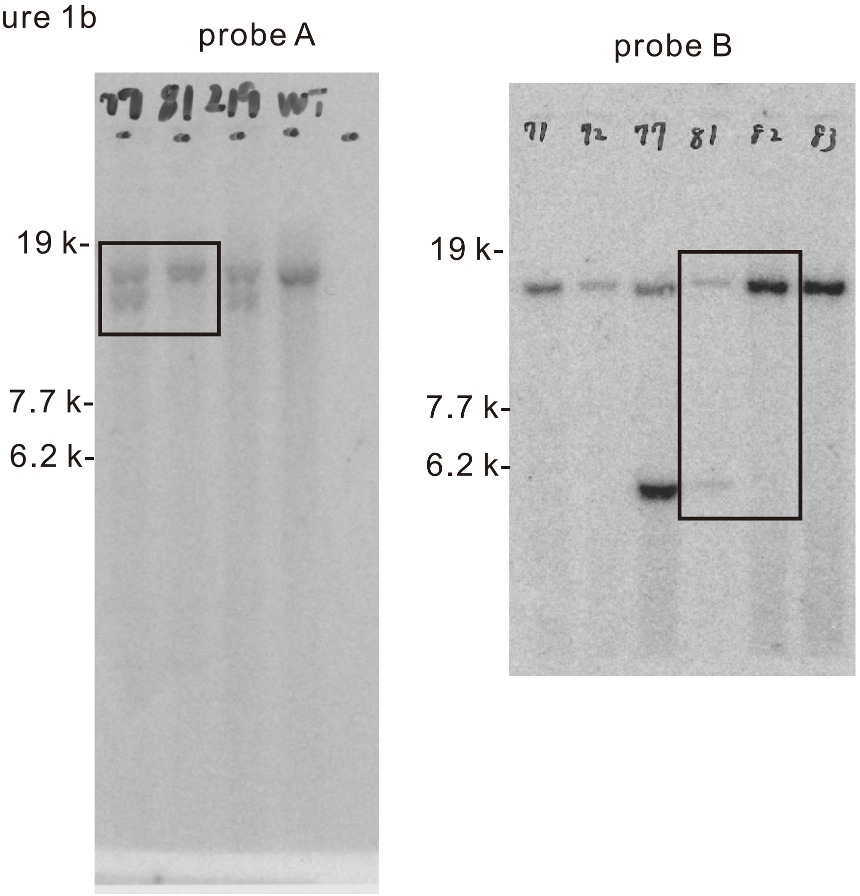

Figure 1c

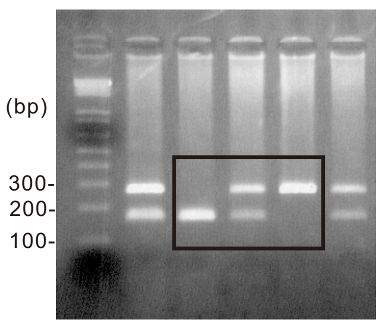

Figure 1d

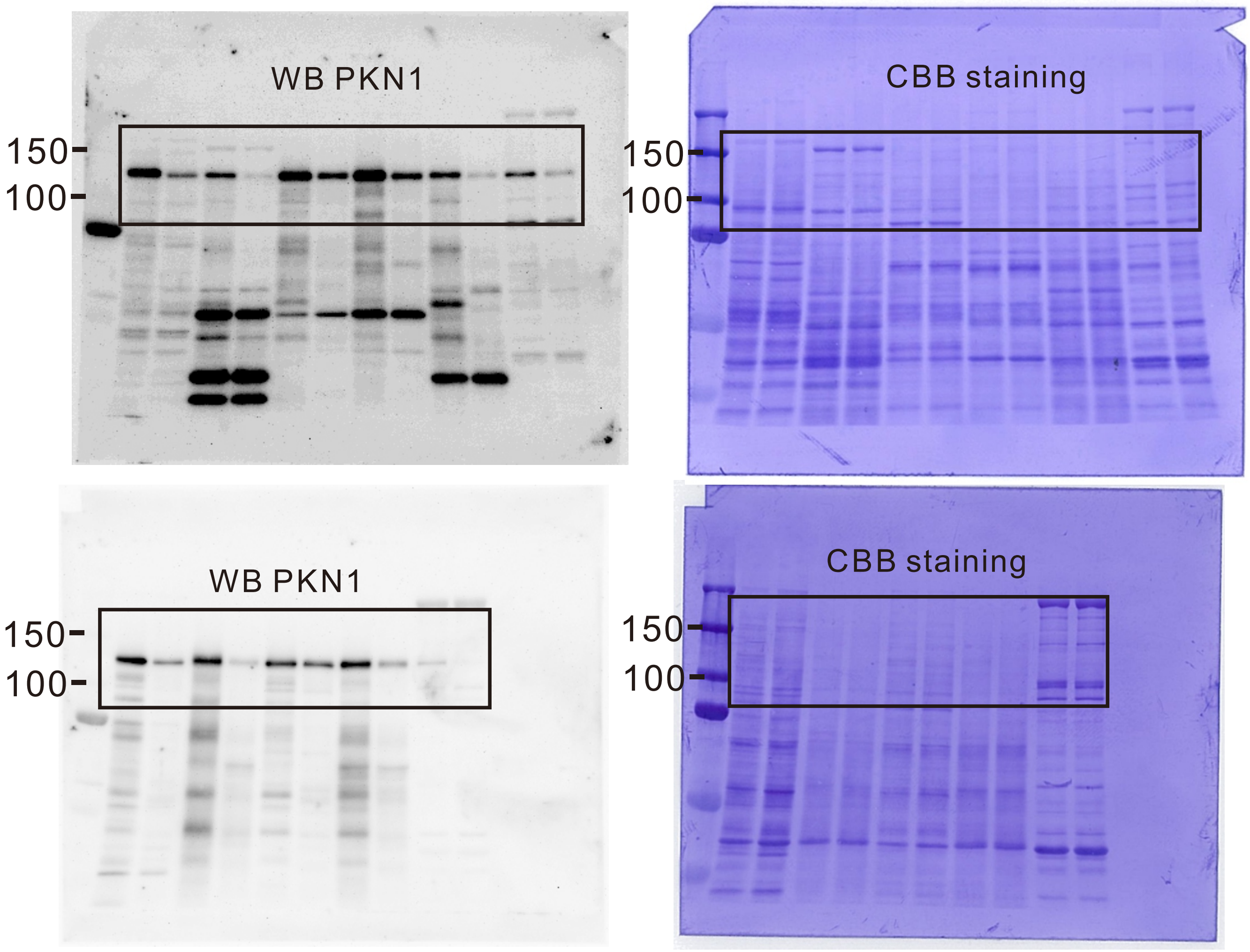

Figure 1f

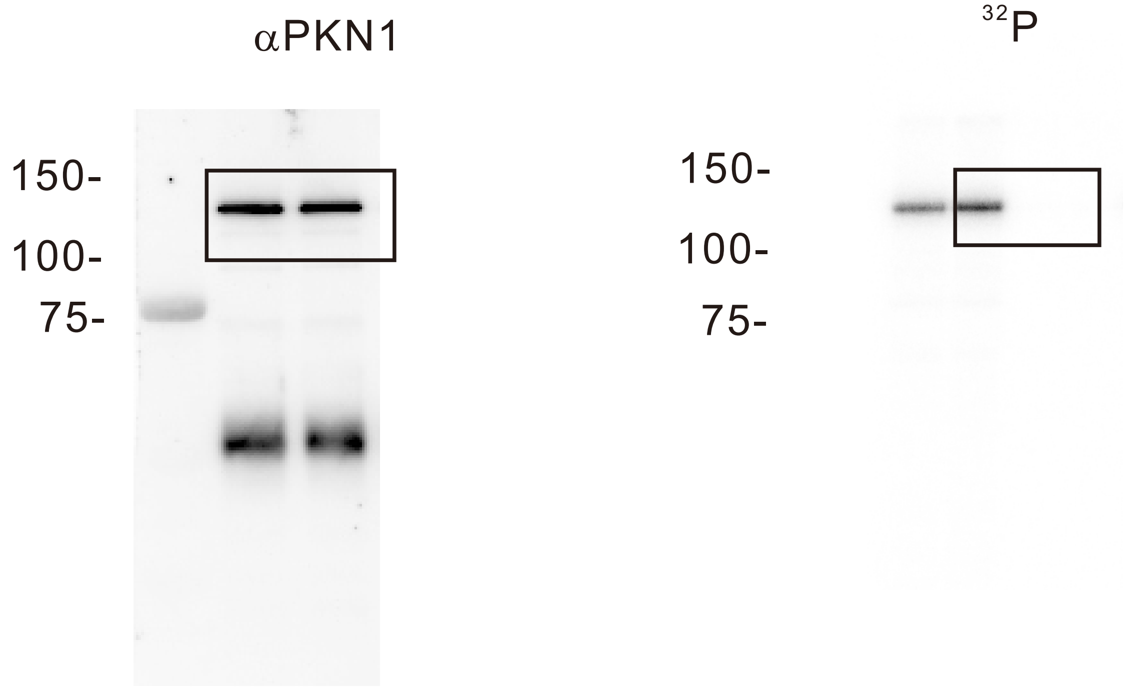

Figure 1g

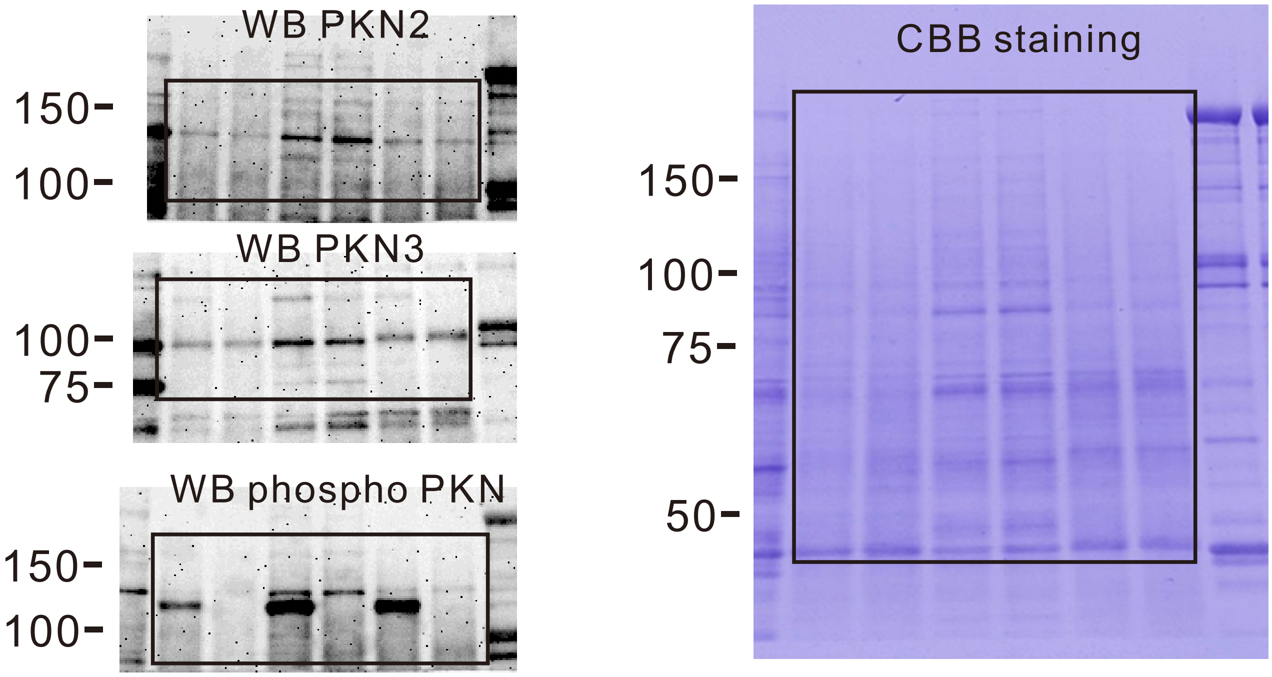

Supplement: Supplementary file 1 — Supplementary Information [file 41598_2017_7936_MOESM1_ESM.pdf]
